# A Self-Powered and Low Pressure Loss Gas Flowmeter Based on Fluid-Elastic Flutter Driven Triboelectric Nanogenerator

**DOI:** 10.3390/s20030729

**Published:** 2020-01-28

**Authors:** Trung Kien Phan, Song Wang, Yan Wang, He Wang, Xiu Xiao, Xinxiang Pan, Minyi Xu, Jianchun Mi

**Affiliations:** 1 Marine Engineering College, Dalian Maritime University, Dalian, 116026, China; trungkienkdt@gmail.com (T.K.P.); wangsong1234@dlmu.edu.cn (S.W.); wangyanme@dlmu.edu.cn (Y.W.); wanghe720815@163.com (H.W.); xiaoxiu@dlmu.edu.cn (X.X.); panxx@dlmu.edu.cn (X.P.); 2 Marine Engineering College, Vietnam Maritime University, Haiphong 180000, Vietnam; 3 School of Electronics and Information technology, Guangdong Ocean University, Zhanjiang 524088, China; 4 College of Engineering, Peking University, Beijing 100871, China

**Keywords:** triboelectric nanogenerator, self-powered sensor, aero-elastic flutter

## Abstract

A self-powered and low pressure loss gas flowmeter is presently proposed and developed based on a membrane’s flutter driven triboelectric nanogenerator (TENG). Such a flowmeter, herein named “TENG flowmeter”, is made of a circular pipe in which two copper electrodes are symmetrically fixed and a nonconductive, thin membrane is placed in the middle plane of the pipe. When a gas flows through the pipe at a sufficiently high speed, the membrane will continuously oscillate between the two electrodes, generating a periodically fluctuating electric voltage whose frequency can be easily measured. As demonstrated experimentally, the fluctuation frequency (*f_F_*) relates linearly with the pipe flow mean velocity (*U_m_*), i.e., *f_F_* ∝ *U_m_*; therefore, the volume flow rate *Q* (=*U_m_* × *A*) = C_1_*f_F_* + C_2_, where C_1_ and C_2_ are experimental constants and *A* is the pipe cross-sectional area. That is, by the TENG flowmeter, the pipe flow rate *Q* can be obtained by measuring the frequency *f_F_*. Notably, the TENG flowmeter has several advantages over some commercial flowmeters (e.g., vortex flowmeter), such as considerable lower pressure loss, higher sensitiveness of the measured flow rate, and self-powering. In addition, the effects of membrane material and geometry as well as flow moisture on the flowmeter are investigated. Finally, the performance of the TENG flowmeter is demonstrated.

## 1. Introduction

A flowmeter is an important device installed within pipe systems for various industrial, marine, and civil applications such as blower, fresh air supply, room air conditioning systems, and inert gas transfer or supply. This device is utilized to measure the flow rate and monitor the working status of the pipe system. At present, the common flowmeters are in the form of magnetic [1,2], ultrasonic [3,4], thermal, orifice plate and vortex meter [5,6], coriolis [7,8], and turbine type [9,10]. The typical three types of flowmeter used today are ultrasonic, coriolis, and vortex ones. Among them, the vortex flowmeter based on the vortex shedding frequency is currently applied in a wide range of piping system because it is not sensitive to physical properties of the fluid and can be used for any medium that is not appropriate for ultrasonic or coriolis devices. Like other flowmeters, the vortex one usually needs a battery for powering. In addition, a pipe system has multiple branch pipes which require flowmeters for monitoring flow rates. The piping with numerous flowmeters powered by battery will inevitably cause an environmental issue, inconvenience of periodical maintenance, and high cost. Therefore, it is significant to develop a self-powered flowmeter with no need of external power supply.

Recently, the triboelectric nanogenerator (TENG) was developed as a device that can efficiently convert mechanical energy from environmental powers, e.g., mechanical vibration, wind energy, tidal energy, and water wave energy, into electricity [11,12,13,14,15,16,17]. The current is alternately generated in the TENG device based on the coupling of triboelectrification effect and electrostatic induction.

Additionally, by utilizing the generated electrical signals, self-powered active sensors based on TENG have been developed for detecting motion, pressure, resonant, sleep monitoring, ion concentration, temperature, tilt of direction, and angle [18,19,20,21,22,23,24,25,26,27]. In particular, various models have been studied for self-powered sensors measuring wind speed and direction [28,29,30]. These studies have shown that the flow-induced vibration of membrane self-powered TENG sensors have the advantages of a simple structure and low-cost compared with other forms of self-powered sensors. Yang et al. [28] developed a self-powered sensor based on a single-electrode TENG to detect the wind speed and direction. The wind speed was determined by measuring the magnitude of the current which is generated by the vibration of the ethylene-propylene (FEP) membrane inside a cuboid acrylic tube. Similarly, Su et al. [29] reported the excellent linear relationship between the wind speed and output current in a free-standing mode TENG structure. Later, Xu et al. [30] proposed that wind speed could be calculated based on the frequency of current/voltage output in an aero-elastic flutter structure. For the pipe systems, the flow can produce the flow-induced vibration of membrane, thus the flow rate can be determined through the vibration frequency. However, those previous flow-induced TENG sensors only focused on the cuboid structures that cannot easily integrate with any pipe systems. Furthermore, there is no information available about the pressure loss of these TENG sensors, which is important for the operation of a pipe system.

To address the above deficits, the present study aimed to develop a self-powered flowmeter (TENG flowmeter) based on TENG for measuring and monitoring the gas flow rate in a pipe system. Specifically, the main objectives of the present study were four-fold:

(1) To approve the linear relationship between the flutter frequency of a membrane and the pipe flow rate;
(2) To investigate the effects of the membrane material and dimension as well as the humidity of airflow on the performance of TENG flowmeter;
(3) To compare the sensitivity and pressure loss of a TENG flowmeter with those of the commercial vortex flowmeter; and
(4) To demonstrate the TENG flowmeter’s performance.

## 2. Structure and Working Principle of the TENG Flowmeter

**Figure 1:**
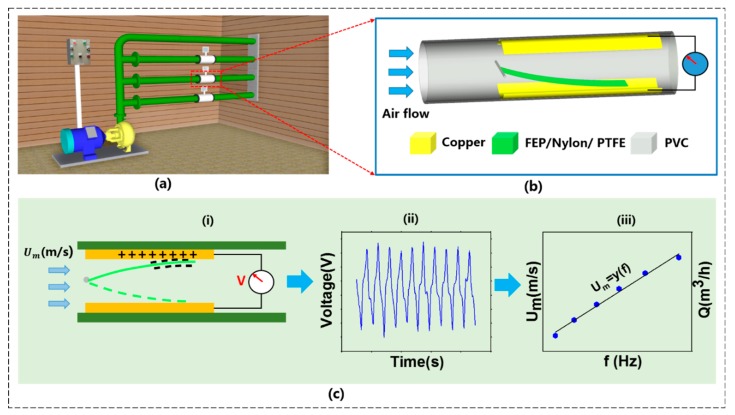
(**a**) Schematic diagram of the fabricated triboelectric nanogenerator (TENG) flowmeter for gas flow rate measurement, (**b**) TENG flowmeter structure, (**ci**) electric harvester principle of the TENG flowmeter, (**cii**) voltage output to time domain, (**ciii**) linear fitting relationship between frequency and gas flow velocity.

Figure 1a displays several TENG flowmeters that are connected with gas pipes. Figure 1b shows the detailed structure of the present TENG flowmeter that consists of three basic components: (1) A straight, circular transparent polymerizing vinyl chloride (PVC) pipe with 30-mm inner diameter and 250-mm length; (2) two conductive copper foil electrodes deposited inside the pipe; (3) a flexible, thin, rectangular membrane in which one end is fixed by a shedder bar in the middle of the pipe and another end is free. The electrodes have a fixed thickness of 0.03 mm, which is thin enough to hardly affect the resistance of gas flow in the pipe; their length and width are designed larger than the size of the membrane to enable the membrane to completely contact with the electrodes when the membrane is vibrating. The free end of the membrane is able to flap to contact with the two electrodes due to the flow-induced vibration. Previously, FEP [28], polydimethylsiloxane (PDMS) [13], Kapton [31], and PVC nanofiber [32] were employed as the membrane material of TENG flutter-driven structures. In order to simplify the structure and save costs, a nonconductive polytetrafluoroethylene (PTFE) membrane was selected for this TENG flowmeter.

The working principle of the TENG flowmeter is based on a triboelectric nanogenerator, where free electrons flow in the external circuit due to triboelectrification and electrostatic induction phenomenon when two different triboelectric materials contact each other alternatively. The charge transfer process is schematically depicted in Figure 1ci. In the initial state, the membrane is located on the bottom electrode due to the gravity for the present case, in which there is no current. When a flow occurs through the pipe at a sufficiently high velocity, the membrane moves up and down periodically due to the flow-induced vibration effect. As the membrane starts to contact with one electrode, some free electrons will be transferred from the electrode to the membrane. The membrane is negatively charged while the electrode is positively charged as the PTFE material is more triboelectrically negative than the copper. When the negatively charged PTFE membrane moves away from one of the copper electrodes, the electric potential difference between the two electrodes will be generated, driving free electrons to flow from one electrode to another through the external circuit. Thus, the flow-induced membrane flutter generates an alternating current in the external circuit. The continuously periodic flutter makes free electrons flow through the external circuit, thus producing the periodic wavy voltage shown in Figure 1cii. Notably, the frequency of the electrical output signal is equivalent to the membrane flutter frequency. To obtain this frequency, the instantly wavy electric signal is converted to that in the frequency domain by the Fast Fourier Transform (FFT) algorithm. It is found that the frequency (*f_F_*) has a linear relationship with the pipe flow mean velocity (*U_m_*), i.e., *f_F_* ∝ *U_m_*, as shown in Figure 1ciii. It follows that the volume flow rate can be calculated as *Q* (= *U_m_* × *A*) = C_1_*f_F_* + C_2_, where C_1_ and C_2_ are experimental constants and *A* is the pipe cross-sectional area. That is, the pipe flow rate *Q* can be obtained by measuring the frequency *f_F_*. It is worth noting that, with a constant flow rate, the fluttering frequency varies with a specific membrane, which suggests that C_1_ and C_2_ depend on the membrane parameter. The values of constants *C_1_* and *C_2_* are obtained by the linear fitting method. Hence, considering the membrane motion being transformed into the electrical power, this simple structure can be used as a self-powered flowmeter.

To summarize, the TENG flowmeter is made of a circular pipe in which two copper electrodes are symmetrically fixed and a nonconductive, thin membrane is placed in the middle plane of the pipe. When a flow takes place through the pipe at a sufficiently high speed, the membrane will continuously oscillate between the two electrodes, generating a periodically wavy electric voltage whose frequency is proportional linear to the pipe flow rate and so it can be measured for the latter.

## 3. Experimental Details

### 3.1. Fabrication of the TENG Flowmeter

The TENG flowmeter is fixed in a circular, transparent PVC pipe having an inner diameter of *D* = 30 mm and a length of 250 mm. The two thin, copper films as side electrodes are 25 mm in width and 200 mm in length. The selected conductive copper electrodes are self-adhesive copper foil which can be firmly attached to the PVC pipe and other different substrates. The front and rear sections of the pipe are drilled holes for measurements of differential pressure and air humidity. A flexible, thin, rectangular film is used as the dielectric membrane with its length, thickness, and width being represented by *L*, *h,* and *W*. One end of membrane is fixed centrally in the pipe. To investigate the effect of a material on the performance of the TENG flowmeter, three membrane materials, i.e., PTFE, nylon, and FEP, with 0.05 mm in thickness were used. Moreover, to examine the effect of membrane dimension, the PTFE membrane was used whose length varied from 40 mm to 90 mm while its width changed between 10 mm and 18 mm. Note that each of these lengths could make the membrane contact the electrodes.

### 3.2. Experimental Setup

**Figure 2:**
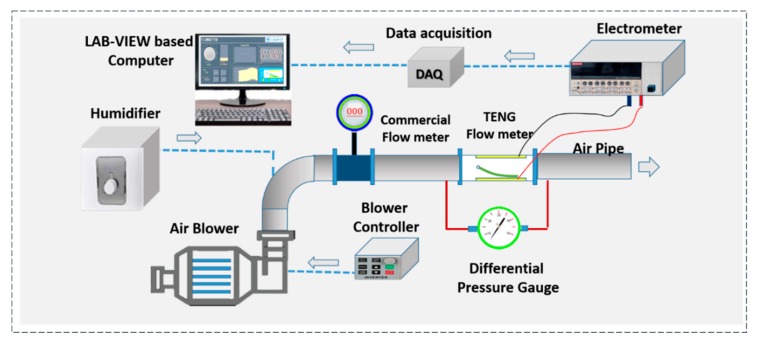
TENG flowmeter experimental apparatus.

The experimental apparatus was as shown in Figure 2. The airflow through the pipe was generated by the electric centrifugal fan motor at room temperature. To control the airflow rate of the fan, an electric frequency inverter driver was used and the frequency was set over a range of 0–50 Hz. In addition, a gas digital vortex commercial flowmeter was installed upstream of the pipeline to measure the flow rate. The mean velocity *U_m_* was specified by *U_m_ = Q*/(*πD*^2^/4), where *Q* is the volume flow rate measured by the flowmeter. For measuring open-circuit voltage and short-circuit current of the TENG flowmeter, the two electrodes were connected to an electrometer (Keithley 6514). The electric signals were then transferred to a data acquisition (DAQ) board (National Instruments, DAQ–9174) for acquisition, and were calculated in time intervals by the LabView^®^ based computer. The gas flow rate measured by the TENG flowmeter was calculated by the signal waveform frequency which was converted from electric voltage by the Matlab software based on FFT algorithm. The pressure drop of the TENG flowmeter was measured by a differential pressure gauge (Testo 510). A commercial humidifier was used to increase the air humidity in the pipe up from 75% to 95%. In this experiment, the flow rate of normal air was used as the working fluid for developing the TENG flowmeter. Significantly, the membrane flutter was a typical fluid–structure interaction phenomenon and the flutter frequency was closely related to gas physical properties, such as gas density and viscosity. In theory, the working principle of the TENG flowmeter is the same when measuring other gases. The linear relationship between the electrical signal frequency and the flow velocity still holds, but the specific linearity depends on different gases. Therefore, when any other gas was used as the measured gas, the TENG flowmeter needed a calibration before use.

## 4. Characterizing the TENG Flowmeter

The operation of the TENG flowmeter relies on the periodical flutter of the rectangular membrane, which can be considered as a cantilevered flat plate with length (*L*), thickness (*h*), and width (*W*). The vibrating membrane may be bent, folded, twisted, or waved under the flow-induced vortex [33,34,35]. The equation of motion of the elastic plate is given below by Euler–Bernoulli beam theory (Equation (1)), which could also apply to the motion of membrane in our TENG flowmeter:

(1) $$\rho_{p}\frac{\partial^{2}Y}{\partial T^{2}}+K\frac{\partial^{4}Y}{\partial X^{4}}=\langle \Delta P\rangle_{z}$$

where *ρ*_p_ is membrane material density, *K* is the flexural rigidity, Δ*P* is the pressure difference between two sides of the membrane, 〈…〉_Z_ denotes the average along the spanwise direction for −*W*/2 < z < *W*/2 [36,37,38,39], and *K* = *Eh*^3^/12[(1 − v^2^)] with *E* being Young’s modulus (tensile elastic modulus) and v being Poisson’s ratio [40]. Accordingly, the characteristics of TENG flowmeter were studied by exploring the effects of various factors of the membrane.

### 4.1. Effects of Various Factors on the Flutter Frequency of a Membrane

#### 4.1.1. Effect of Membrane Material

**Figure 3:**
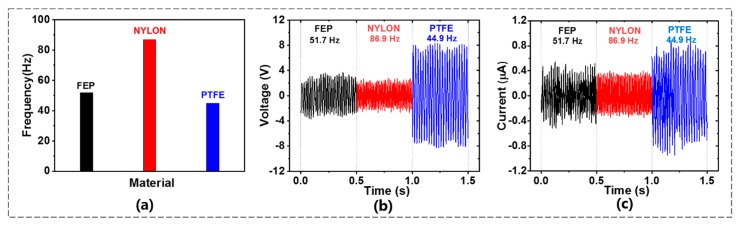
(**a**) Frequency, (**b**) voltage, (**c**) current of TENG flowmeter with various membrane materials.

To study the effect of membrane material on the output performance of TENG flowmeter, three membrane materials (i.e., PTFE, FEP, and nylon) were tested on the TENG flowmeter. These membranes use the same size with the length *L* = 60 mm, width *W* = 16 mm, and thickness *h* = 0.05 mm. As shown in Figure 3a–c, although the airflow velocity was the same (*U_m_* = 7.86 m/s), the fluttering frequency of the nylon membrane (86.9 Hz) was larger than those of PTFE (44.9 Hz) and FEP (51.7 Hz). Thus, it is suggested that the measurement range and the sensitivity of the TENG flowmeter with nylon membrane may be higher. However, the PTFE membrane produced much higher voltage and current than the FEP and nylon membranes, perhaps due to its higher electron affinity [41]. It is known that the flutter frequency of the membrane is determined by the frequency of the voltage signal. The application of PTFE material allowed is to accurately determine the signal frequency with a common electrometer, especially for the flow case with high relative humidity. Therefore, the PTFE material was used in the following.

#### 4.1.2. Effect of Membrane Dimension

**Figure 4:**
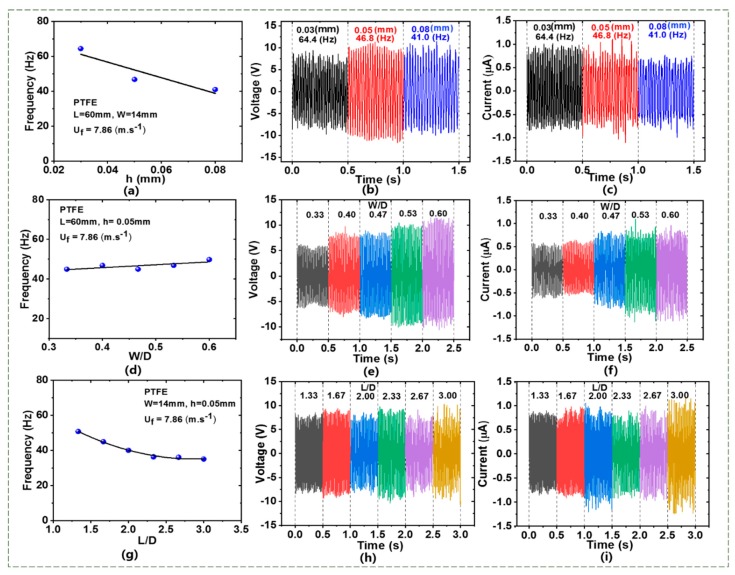
Flutter frequency and electrical characteristics of TENG flowmeter with PTFE membrane at the same airflow velocity of 7.86 m/s. (**a**) Frequency, (**b**) voltage, (**c**) current with different thickness. (**d**) Frequency, (**e**) voltage, (**f**) current with different width. (**g**) Frequency, (**h**) voltage, (**i**) current with different length.

To study the effect of membrane thickness (*h*) on the performance of the TENG flowmeter, Figure 4a–c shows the flutter frequency of the PTPE membrane together with the time-instant voltages and currents at *h* = 0.03–0.08 mm and *W/D* = 0.46 and *L/D* = 2 at the same airflow velocity of 7.86 m/s. Evidently, as the thickness increased, the frequency decreased due to the reduced flexibility. Additionally, the decrease in the flutter frequency reduced the electric charge rate of the membrane to the electrodes, leading to a decrease in the short-circuit current, consistent with the previous studies [42,43]. Moreover, it was shown that the open-circuit voltage for *h* = 0.05 mm was higher than those for *h* = 0.03 mm and 0.08 mm. This result may be due to a larger contact area at *h* = 0.05 mm [33].

Figure 4d–f shows the membrane’s flutter frequency as well as the time-instant voltages and currents at *W/D* = 0.33–0.6 and *h* = 0.05 mm and *L/D* = 2 at *U_m_* = 7.86 m/s to examine the dependence of membrane width (*W*). The widths of *W/D* < 1 were selected so that there was a sufficient space for the membrane to oscillate in the pipe. Evidently, from Figure 4e,f, as the membrane width increased, both the output voltage and the current rose because the effective contact area of the membrane with the electrodes grew. It was also demonstrated that the flutter frequency increased slightly and linearly with increasing the width, see Figure 4d. Of note, as *W* increased from 10 mm to 18 mm, the output voltage rose from 10 V to 22 V while the current grew from 0.5 µA to 0.8 µA.

Figure 4g–i shows the experimental results for the effects of the membrane length (*L*) on the characteristics of the TENG flowmeter. Six different lengths of the PTFE membrane were employed between 40 mm and 90 mm with the same width and thickness (*W* = 14 mm, and *h* = 0.05 mm). All the lengths were larger than the pipe inner diameter (*D*), and so the dimensionless ratios *L/D* = 1.33–3 allowed the membrane to contact well with the electrodes when fluttering. Figure 4g demonstrates that, as *L* increased, the flutter frequency significantly dropped between *L/D* = 1.33 and *L/D* = 2.33 but decreased little from *L/D* = 2.33 to *L/D* = 3. The former observation is consistent with that from Xu et al. [44] who investigated a free membrane-induced flapping jet. The latter observation may be associated with the fact that, since the membrane is confined in the pipe, when *L* is increased beyond 60 mm, the contact length of the membrane will increase, see Supplementary Materials Figure S1. Moreover, as *L* was increased from 40 mm to 90 mm, the electric output slightly changed, including short-circuit current (about 0.8–1.1 μA) and open-circuit voltage (about 14–16 V), as shown in Figure 4h,i. The results imply that the length of PTFE membrane affects the electric output slightly but the frequency strongly.

#### 4.1.3. Effect of Airflow Moisture

**Figure 5:**
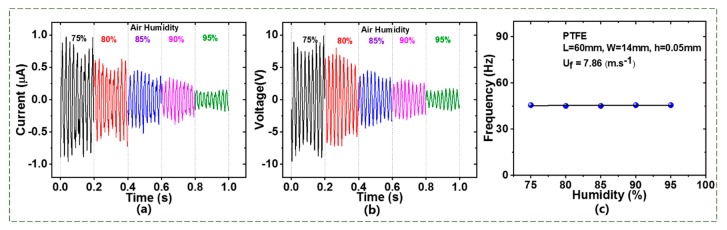
Humidity effect on the (**a**) current, (**b**) voltage, and (**c**) flutter frequency of the TENG flowmeter.

To investigate the effect of relative humidity of air on the TENG flowmeter’s performance, the moist air flow at *U_f_* = 7.86 m/s was fed into the pipe. The relative humidity (RH) of the air was adjusted to increase gradually from 75% to 95%. At each value of humidity, the short-circuit current, open-circuit voltage, and the frequency were measured. As shown in Figure 5a,b, the output current and voltage decreased greatly with increasing the humidity. More specifically, the current decreased from 0.8 to 0.2 (µA) and the voltage decreased from 18 to 2 V. This is likely because, as the humidity rose, the wet air became more conductive, so that the amount of electric charges with the membrane reduced. However, Figure 5c demonstrates that the fluttering frequency did not change at all for varying the airflow humidity, which agrees with the result of Xu. et al. [30]. This also suggests that the TENG flowmeter can efficiently work in various wet gas flows.

### 4.2. Relationship of the Flow Rate and Flutter Frequency

**Figure 6:**
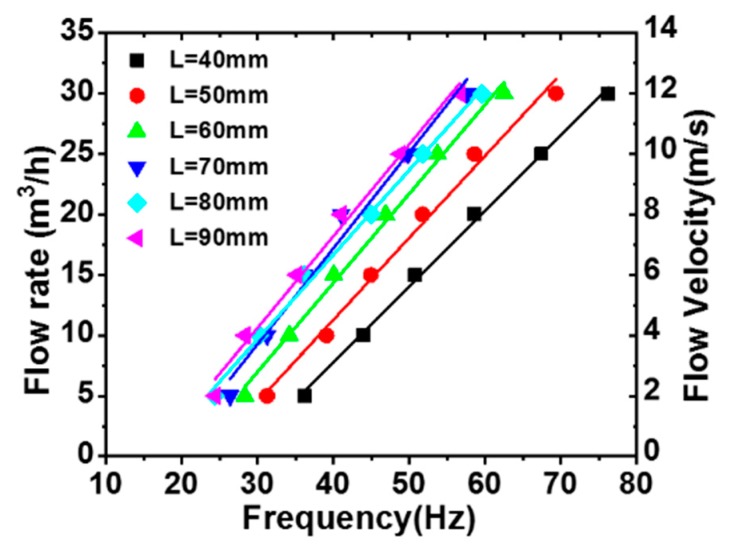
Pipe flow velocity and volume flow rate versus the flutter frequency.

Figure 6 shows the pipe flow rate (*Q*) versus the flutter frequency (*f_F_*) for different length PTFE membranes. Clearly, there was a good linear relationship between *Q* and *f_F_* as *L* changed from 40 mm to 90 mm, i.e., *Q* = C_1_*f_F_* + C_2_, where C_1_ and C_2_ were experimental constants obtained by the linear fitting method. Namely, the flow rate *Q* can be obtained from measuring the fluttering frequency *f_F_* of the membrane. Measurement accuracy is one of the most important parameters for the flowmeter. Worth noting is that the slope of the linear relationship increased from C_1_ = 0.63 to C_1_ = 0.75 as *L* increased from 40 to 90 mm. This indicates that decreasing the length of membrane can increase the measured accuracy of the flow rate of the TENG flowmeter.

Note that the accuracy issue of the present TENG flowmeter is important. To address this issue, the air flow rate measured by the commercial vortex flowmeter installed upstream of the pipeline was used as the reference, and the measurement accuracy (*S*) of the TENG flowmeter can be calculated by

(2) $$S(\%)=\frac{1}{n}\sum \left|\frac{Q-Q_{V}}{Q_{V}}\right|*100\%$$

where *Q_V_* is the actual airflow rate measured by the vortex flowmeter and *n* is the number of measurements. For example, for the TENG flowmeter with membrane length of *L* = 40 mm, *C*_1_ = 0.63, *C*_2_ = –17.32, the calculated accuracy is *S* = 2.8%. In addition, the noise signal was measured when the membrane was stable, as shown in Figure S3. It was found that the magnitude of the noise was about 0.16 V, while the output voltage of the TENG measured at the wind speed of 7 m/s was about 12 V. Thus, the signal/noise ratio *R* ≈ 20log(12/0.16) = 35 dB was very high. Usually, the voltage of the TENG was high enough to distinguish the output current and voltage signals from the noise.

## 5. Demonstration of the TENG Flowmeter

**Figure 7:**
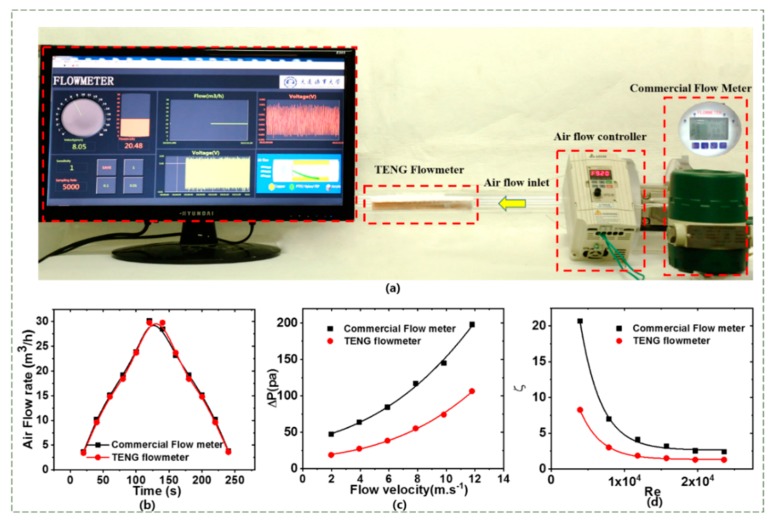
Demonstration of the TENG flowmeter for measuring real-time flow rate. (**a**) Photos of the real-time gas flow rate measurement using TENG flowmeter. (**b**) Comparison of TENG flowmeter and commercial gas flowmeter. (**c**) Pressure loss under different airflow velocities. (**d**) Pressure loss coefficient corresponding to Reynolds number.

The performance of the TENG flowmeter to measure the flow rate through a circular PVC pipe of 30 mm in diameter is demonstrated in Figure 7. The airflow was generated by the electric centrifugal fan motor and measured also by a commercial flowmeter. The flutter frequency of the membrane was equal to the frequency of the fluctuating output voltage (*f*) that can be measured using the short-time FFT technique with the computer interface by Labview program, see Figure 7a. Then, the flow mean velocity *U_m_* was obtained by using the output voltage frequency through the relation *U_m_* = G(*f_F_*) and then the mean airflow rate *Q = U_m_A,* where *A* is the cross-sectional area of the pipe. The display monitor showed the measured *Q* (see Figure 7a and Supplementary Materials Video S1). The measurements of *Q* by the TENG flowmeter were compared with those by the commercial flowmeter in Figure 7b. Evidently, there was almost no difference between the measurements of the two devices. When the airflow rate was changed, the TENG flowmeter performed more sensitively than did the commercial device. For instance, when the airflow rate was reduced from 30 m^3^/h to 25 m^3^/h, the screen displayed almost immediately these flow rates as well as the corresponding velocities, while the commercial flowmeter needed to take some time to show them (see Supplementary Materials Video S1).

The other superior characteristic of the TENG flowmeter is its low pressure loss. To evaluate the pressure loss of the TENG flowmeter during operation, the pressure difference (ΔP) between the inlet and outlet of the TENG flowmeter was measured under various flow velocities of *U_m_* = 1.96 to 11.79 m/s. These measurements were then compared with those for the commercial vortex flowmeter in Figure 7c. Obviously, the pressure loss of the TENG flowmeter was much less than the commercial flowmeter. For instance, at *U_m_* = 1.96 m/s, the pressure loss of the former (ΔP = 18.9 Pa) was about 60% lower than that of the latter (ΔP = 47.3 Pa). As the airflow velocity increased to *U_m_* = 11.79 m/s, the pressure loss of the TENG flowmeter grew quickly (ΔP = 106 Pa) but was still 40% lower than the commercial one (ΔP = 198 Pa). It was hence proved that the TENG flowmeter had a much lower pressure loss compared with the commercial flowmeter.

The coefficient (ξ) of pressure loss for both flowmeters can be calculated below:

(3) $$\Delta P=\xi \frac{\rho_{a}.U_{m}^{2}}{2}$$

where ρ_a_ is density of the air. Figure 7d shows that, as the Reynolds number *Re* (= *U_m_D*/ν) increased, the pressure loss coefficients of both the TENG and commercial flowmeters dropped. Very significantly, the coefficient was much lower for the TENG flowmeter, which is important for industrial applications.

Moreover, the pressure drop of the TENG flowmeter at various PTFE membrane widths was measured at the airflow velocity of 7.86 m/s and is shown in Supplementary Figure S2a. The pressure loss ΔP increased approximately linearly with increasing the membrane width *W*. For example, ΔP increased from 47 Pa to 78 Pa when increasing *W* from 10 mm to 18 mm. For a wider membrane, ΔP increased due to more airflow friction resistance. Similarly, we also measured the pressure drop with varying membrane length *L*. It was interesting to find that ΔP varied little around 62 Pa when *L* was increased from 40 mm to 90 mm at the same airflow rate (see Supplementary Figure S2b). This proved that *L* had little effect on the pressure loss of the TENG flowmeter.

## 6. Conclusions

We proposed and developed a simple and practical design for a self-powered and low pressure loss gas TENG flowmeter. The fabricated TENG flowmeter consisted of two copper electrode layers and a PTFE membrane in a circular, acrylic pipe. The effects of membrane material, membrane dimension, and gas humidity on the performance of the TENG flowmeter were experimentally examined. The experimental results showed that the linear relationship between the fluttering frequency and gas flow rate worked well for different membranes. The comparison of the measurement results between the present TENG flowmeter and the commercial vortex flowmeter indicated that the newly designed flowmeter had a good measurement accuracy. In addition, the low pressure loss of the TENG flowmeter was proven, as well. Thus, the TENG flowmeter can be used as a self-powered, real-time, monitoring flowmeter for measuring the gas flow velocity and, thus, the flow rate through any round pipe system.

## Supplementary Materials

The following are available online at https://www.mdpi.com/1424-8220/20/3/729/s1. Figure S1: Fluttering images of PTFE membrane of thickness h = 0.05 mm, width W = 14 mm, and Um = 7.86 m/s with various length from 40 to 90 mm by a high speed camera. Figure S2: (a) Pressure loss of TENG flowmeter of with various widths, (b) lengths. Figure S3: The comparison of noise signal measured at the membrane is stable, and the sensor signal when the membrane is fluttering at the wind speed of 7 m/s. Video S1: Demonstration of the TENG flowmeter for measuring flow rate.

## References

[1] Sahu S., Prajapati A., Kumar M., Bhattacharyay R. (2019). Development of an optimised magnetic field source for flowmeter applications. Flow Meas. Instrum..

[2] Salustiano Martim A.L.S., Dalfré Filho J.G., De Lucca Y.d.F.L., Borri Genovez A.I. (2019). Electromagnetic flowmeter evaluation in real facilities: Velocity profiles and error analysis. Flow Meas. Instrum..

[3] Furuichi N. (2013). Fundamental uncertainty analysis of flowrate measurement using the ultrasonic Doppler velocity profile method. Flow Meas. Instrum..

[4] Lynnworth L.C., Liu Y. (2006). Ultrasonic flowmeters: Half-century progress report, 1955–2005. Ultrasonics.

[5] Pankanin G.L. (2005). The vortex flowmeter: Various methods of investigating phenomena. Meas. Sci. Technol..

[6] Zhang H., Huang Y., Sun Z. (2006). A study of mass flow rate measurement based on the vortex shedding principle. Flow Meas. Instrum..

[7] Wang T., Baker R. (2014). Coriolis flowmeters: A review of developments over the past 20 years, and an assessment of the state of the art and likely future directions. Flow Meas. Instrum..

[8] Groenesteijn J., Droogendijk H., Wiegerink R.J., Lammerink T.S.J., Lötters J.C., Sanders R.G.P., Krijnen G.J.M. (2014). Parametric amplification in a micro Coriolis mass flow sensor. J. Appl. Phys..

[9] Džemić Z., Širok B., Bizjan B. (2018). Turbine flowmeter response to transitional flow regimes. Flow Meas. Instrum..

[10] Lee B., Cheesewright R., Clark C. (2004). The dynamic response of small turbine flowmeters in liquid flows. Flow Meas. Instrum..

[11] Xu M., Wang P., Wang Y.-C., Zhang S.L., Wang A.C., Zhang C., Wang Z., Pan X., Wang Z.L. (2018). A Soft and Robust Spring Based Triboelectric Nanogenerator for Harvesting Arbitrary Directional Vibration Energy and Self-Powered Vibration Sensing. Adv. Energy Mater..

[12] Wang Z.L., Jiang T., Xu L. (2017). Toward the blue energy dream by triboelectric nanogenerator networks. Nano Energy.

[13] Seol M.-L., Woo J.-H., Jeon S.-B., Kim D., Park S.-J., Hur J., Choi Y.-K. (2015). Vertically stacked thin triboelectric nanogenerator for wind energy harvesting. Nano Energy.

[14] Yong H., Chung J., Choi D., Jung D., Cho M., Lee S. (2016). Highly reliable wind-rolling triboelectric nanogenerator operating in a wide wind speed range. Sci Rep-UK.

[15] Xia K., Tang H., Fu J., Tian Y., Xu Z., Lu J., Zhu Z. (2020). A high strength triboelectric nanogenerator based on rigid-flexible coupling design for energy storage system. Nano Energy.

[16] Xia K., Zhu Z., Fu J., Li Y., Chi Y., Zhang H., Du C., Xu Z. (2019). A triboelectric nanogenerator based on waste tea leaves and packaging bags for powering electronic office supplies and behavior monitoring. Nano Energy.

[17] Xia K., Zhu Z., Zhang H., Du C., Fu J., Xu Z. (2019). Milk-based triboelectric nanogenerator on paper for harvesting energy from human body motion. Nano Energy.

[18] Wu Z., Ding W., Dai Y., Dong K., Wu C., Zhang L., Lin Z., Cheng J., Wang Z.L. (2018). Self-Powered Multifunctional Motion Sensor Enabled by Magnetic-Regulated Triboelectric Nanogenerator. ACS Nano.

[19] Lee K.Y., Yoon H.-J., Jiang T., Wen X., Seung W., Kim S.-W., Wang Z.L. (2016). Fully Packaged Self-Powered Triboelectric Pressure Sensor Using Hemispheres-Array. Adv. Energy Mater..

[20] Chen J., Zhang C., Xuan W., Yu L., Dong S., Xie Y., Yin W., Luo J. (2019). Triboelectric Nanogenerator-Based Self-Powered Resonant Sensor for Non-Destructive Defect Detection. Sensors.

[21] Roh H., Kim I., Yu J., Kim D., Chen J., Zhang C., Xuan W., Yu L., Dong S., Xie Y. (2018). Self-Power Dynamic Sensor Based on Triboelectrification for Tilt of Direction and Angle. Sensors.

[22] Jing Q., Zhu G., Wu W., Bai P., Xie Y., Han R.P.S., Wang Z.L. (2014). Self-powered triboelectric velocity sensor for dual-mode sensing of rectified linear and rotary motions. Nano Energy.

[23] Wang P., Pan L., Wang J., Xu M., Dai G., Zou H., Dong K., Wang Z.L. (2018). An Ultra-Low-Friction Triboelectric–Electromagnetic Hybrid Nanogenerator for Rotation Energy Harvesting and Self-Powered Wind Speed Sensor. ACS Nano.

[24] Chen C., Wen Z., Wei A., Xie X., Zhai N., Wei X., Peng M., Liu Y., Sun X., Yeow J.T.W. (2019). Self-powered on-line ion concentration monitor in water transportation driven by triboelectric nanogenerator. Nano Energy.

[25] Peng M., Wen Z., Xie L., Cheng J., Jia Z., Shi D., Zeng H., Zhao B., Liang Z., Li T. (2019). 3D Printing of Ultralight Biomimetic Hierarchical Graphene Materials with Exceptional Stiffness and Resilience. Adv. Mater..

[26] Xie L., Chen X., Wen Z., Yang Y., Shi J., Chen C., Peng M., Liu Y., Sun X. (2019). Spiral Steel Wire Based Fiber-Shaped Stretchable and Tailorable Triboelectric Nanogenerator for Wearable Power Source and Active Gesture Sensor. Nano-Micro Letters.

[27] Xie X., Zhang Y., Chen C., Chen X., Yao T., Peng M., Chen X., Nie B., Wen Z., Sun X. (2019). Frequency-independent self-powered sensing based on capacitive impedance matching effect of triboelectric nanogenerator. Nano Energy.

[28] Yang Y., Zhu G., Zhang H., Chen J., Zhong X., Lin Z.-H., Su Y., Bai P., Wen X., Wang Z.L. (2013). Triboelectric Nanogenerator for Harvesting Wind Energy and as Self-Powered Wind Vector Sensor System. ACS Nano.

[29] Su Y., Xie G., Xie T., Zhang H., Ye Z., Jing Q., Tai H., Du X., Jiang Y. (2016). Wind energy harvesting and self-powered flow rate sensor enabled by contact electrification. J. Phys. D: Appl. Phys..

[30] Xu M., Wang Y.-C., Zhang S.L., Ding W., Cheng J., He X., Zhang P., Wang Z., Pan X., Wang Z.L. (2017). An aeroelastic flutter based triboelectric nanogenerator as a self-powered active wind speed sensor in harsh environment. Extreme Mech. Lett..

[31] Wang S., Mu X., Wang X., Gu A.Y., Wang Z.L., Yang Y. (2015). Elasto-Aerodynamics-Driven Triboelectric Nanogenerator for Scavenging Air-Flow Energy. ACS Nano.

[32] Phan H., Shin D.M., Heon Jeon S., Young Kang T., Han P., Han Kim G., Kook Kim H., Kim K., Hwang Y.H., Won Hong S. (2017). Aerodynamic and aeroelastic flutters driven triboelectric nanogenerators for harvesting broadband airflow energy. Nano Energy.

[33] Liu F., Cai J., Zhu Y., Tsai H.M., Wong A.S.F. (2001). Calculation of Wing Flutter by a Coupled Fluid-Structure Method. J. Aircr..

[34] Huang W.-X., Sung H.J. (2010). Three-dimensional simulation of a flapping flag in a uniform flow. J. Fluid Mech..

[35] Argentina M., Mahadevan L. (2005). Fluid-flow-induced flutter of a flag. Proc. Natl. Acad. Sci. USA.

[36] Eloy C., Souilliez C., Schouveiler L. (2007). Flutter of a rectangular plate. J. Fluids Struct..

[37] Connell B.S.H., Yue D.K.P. (2007). Flapping dynamics of a flag in a uniform stream. J. Fluid Mech..

[38] Virot E., Amandolese X., Hémon P. (2013). Fluttering flags: An experimental study of fluid forces. J. Fluids Struct..

[39] Eloy C., Lagrange R., Souilliez C., Schouveiler L. (2008). Aeroelastic instability of cantilevered flexible plates in uniform flow. J. Fluid Mech..

[40] Perez M., Boisseau S., Gasnier P., Willemin J., Reboud J.L. (2015). An electret-based aeroelastic flutter energy harvester. Smart Mater. Struct..

[41] Wang Z.L. (2013). Triboelectric Nanogenerators as New Energy Technology for Self-Powered Systems and as Active Mechanical and Chemical Sensors. ACS Nano.

[42] Xie Y., Wang S., Lin L., Jing Q., Lin Z.-H., Niu S., Wu Z., Wang Z.L. (2013). Rotary Triboelectric Nanogenerator Based on a Hybridized Mechanism for Harvesting Wind Energy. ACS Nano.

[43] Wang S., Mu X., Yang Y., Sun C., Gu A.Y., Wang Z.L. (2015). Flow-Driven Triboelectric Generator for Directly Powering a Wireless Sensor Node. Adv. Mater..

[44] Xu M., Wu M., Mi J. (2019). A new type of self-excited flapping jets due to a flexible film at the nozzle exit. Exp. Therm Fluid Sci..

