# Supplementary material for: A Self-Powered and Low Pressure Loss Gas Flowmeter Based on Fluid-Elastic Flutter Driven Triboelectric Nanogenerator
Source: Sensors (Basel). 2020 Jan 28;20(3):729. doi: 10.3390/s20030729 (PMC7038380; doi:10.3390/s20030729)
Supplement: Supplementary file 1 [file sensors-20-00729-s001.pdf]

Article

A self-powered and low pressure-loss gas flowmeter based on fluid-elastic flutter driven triboelectric nanogenerator

Trung Kien Phan [1,4], Song Wang [1], Yan Wang [1], He Wang [1], Xiu Xiao [1], Xinxiang Pan [1,2], Minyi Xu [1,*] and Jianchun Mi [1,3,*]

[1] Marine Engineering College, Dalian Maritime University, Dalian, 116026, China
[2] School of Electronics and Information technology, Guangdong Ocean University, Zhanjiang, 524088, China
[3] College of Engineering, Peking University, Beijing, 100871, China
[4] Marine Engineering College, Vietnam Maritime University, Haiphong, 180000, Vietnam
* Correspondence: xuminyi@dlmu.edu.cn (M.X.); jmi@pku.edu.cn (J.M.)

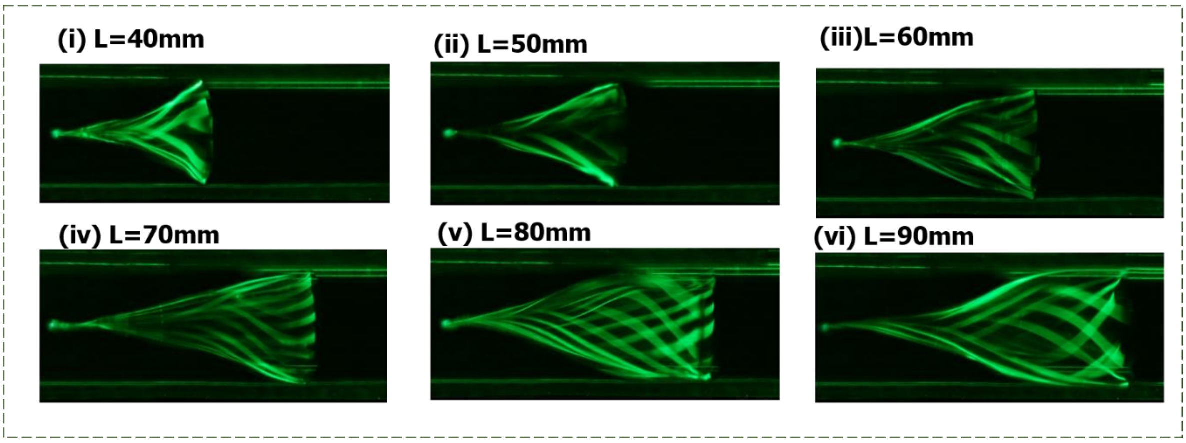

Supplementary Figure S1. Fluttering images of PTFE membrane of thickness h = 0.05 mm, width W = 14 mm and Um = 7.86 m/s with various length from 40 to 90 mm by a high speed camera.

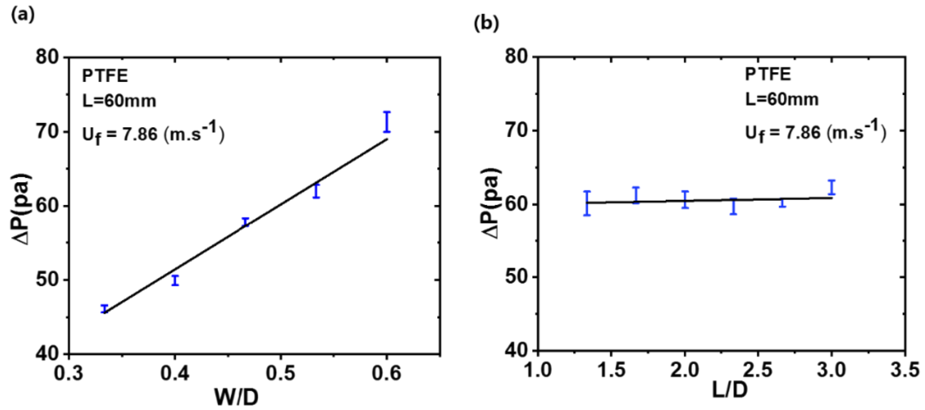

Supplementary Figure S2. (a) Pressure loss of TENG flowmeter of with various widths, (b) lengths.

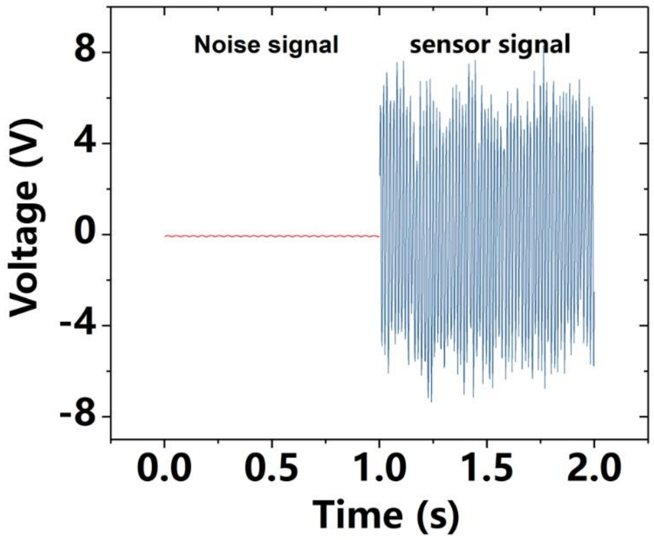

Supplementary Figure S3 The comparison of noise signal measured at the membrane is stable, and the sensor signal when the membrane is fluttering at the wind speed of 7 m/s.
